# Knowledge, Attitude, and Practice of First Aid for Epistaxis Among the General Population in the Jazan Region of Saudi Arabia

**DOI:** 10.7759/cureus.44774

**Published:** 2023-09-06

**Authors:** Naif K Mahzara, Abdullah A Mawkili, Khalid Muafa, Abdulrahman A Aqeel, Nihal Adawi, Alhanouf H Zuqayl, Halah Shayani, Abdulaziz Rajhi, Areej Hakami, Muhammad A Almahdi, Rahf Hakami, Mohammed Muafa, Ibrahim M Dighriri

**Affiliations:** 1 Faculty of Medicine, Jazan University, Jazan, SAU; 2 Department of Otorhinolaryngology, Faculty of Medicine, Jazan University, Jazan, SAU; 3 Department of Medicine and Surgery, Faculty of Medicine, Jazan University, Jazan, SAU; 4 Faculty of Medicine, Jazan University, jazan, SAU; 5 Department of Pharmacy, King Abdulaziz Specialist Hospital, Taif, SAU

**Keywords:** nasal bleeding, nosebleed, jazan, bleeding, first aid, epistaxis

## Abstract

Background

Epistaxis, or nosebleeds, is a widespread medical condition that can be effectively managed with appropriate first aid. Understanding the general public's knowledge and practices about this is crucial.

Objectives

This study sought to evaluate the awareness and practice regarding first aid for epistaxis within the general population of the Jazan region in Saudi Arabia.

Methods

A cross-sectional survey was administered from April through June 2023, using a questionnaire that covered sociodemographic factors, knowledge of epistaxis, first aid practices for epistaxis, and any previous training received. Statistical analysis was performed using SPSS (IBM Corp., Armonk, NY), with chi-square tests to evaluate the variables' associations.

Results

The questionnaire was completed by 622 participants, predominantly females, Saudis, and individuals from the age group of 18 to 25 years. It was found that 60% of the participants had experienced epistaxis, but only 52% had received prior first aid training. Although the majority (91.8%) accurately defined epistaxis, a mere 40.8% correctly identified all the steps for first aid management of epistaxis. There was a notable insufficiency in understanding the causes, risk factors, and appropriate first aid steps. Participants' knowledge was evenly split, with approximately half exhibiting low knowledge (49.70%) and the remainder showing high knowledge (50.30%). Certain sociodemographic factors such as older age (p=0.028), Saudi nationality (p=0.045), and higher education (p=0.001) were linked with more experiences of epistaxis. Conversely, younger age (p=0.002), female gender (p=0.036), single status (p=0.001), prior experience with epistaxis (p=0.001), and higher overall knowledge (p=0.001) were associated with a higher likelihood of having received first aid training.

Conclusions

The study reveals significant gaps in the knowledge and practices of first aid for epistaxis among the general population in the Jazan region. Public awareness campaigns and educational programs are urgently needed, particularly for specific groups. Enhancing first aid knowledge could help alleviate the impacts of epistaxis. Further research is required to develop effective educational interventions.

## Introduction

Epistaxis, or internal nose or nasal cavity bleeding, is a typical medical emergency worldwide in otorhinolaryngology [1]. Although most cases are minor, some can be severe, life-threatening, and indicative of a more serious disease [2]. Epistaxis is classified as anterior or posterior bleeding, with the anterior part of the nose being the most common source, and this classification informs treatment strategies [3]. Various local, systemic, and environmental factors can cause epistaxis, but the primary cause differs depending on the patient's age [4,5]. In 90%-95% of cases, first aid management successfully stops nosebleeds [6]. Conservative, medical, and surgical treatments are also available for epistaxis [4]. In the United States, at least 60% of people experience a nosebleed at some point, and around 6% seek medical help [7].

There needs to be more knowledge on proper first aid management of epistaxis, which often can be effectively treated without a doctor. Studies from the United Kingdom have shown insufficient awareness of first aid management among the general public and healthcare professionals [8]. Research has also found that educating parents about simple management techniques can significantly improve quality-of-life concerns related to epistaxis [9].

In Saudi Arabia, past research has shown that medical students have sufficient knowledge to administer first aid for epistaxis [10]. Other studies in Riyadh and Jeddah have found that school teachers and students are reasonably knowledgeable about management [11,12]. However, there is a noticeable knowledge gap about first aid among the general population, especially in the Jazan region of Saudi Arabia. A limited number of studies have been conducted, reflecting a general lack of awareness about the importance of first aid for epistaxis and its impact on quality of life. This study aimed to evaluate the knowledge and practices of the Jazan region's population concerning first aid for epistaxis and examine the relationship between knowledge, practice level, and various sociodemographic factors.

## Materials and methods

Study design and settings

A cross-sectional study was conducted in the Jazan region between April and June 2023 to assess knowledge, attitude, and practice in regard to epistaxis.

Targeted population

All people in the Jazan region who were 18 years or older and willing to participate were included in the study. People not able to participate or those below 18 years of age were excluded from this study.

Data collection process

Data were collected using an online self-administered questionnaire modified from previous studies [11-13]. The questionnaire contained information about the participants' sociodemographic characteristics, such as age group, sex, nationality, and residence. Furthermore, the questionnaire also included questions about knowledge and practice of first aid for epistaxis among the adult population of Jazan. Participants received a score of "1" for each correct response, while incorrect answers were given a score of "0." Participants with a score less than 50% of the total were classified as having poor knowledge, and those with a score greater than 50% were considered to have good knowledge.

Sampling method and sample size

A convenient sampling method was used. The sample size was estimated to be 440 for this study, using the following formula: 𝑛 = 𝑍²𝑝(1−𝑝) / 𝑑², where *n* = sample size, *Z* = 1.96 (using a 95% confidence level), *p* = 50% (to obtain the largest sample size, as the population distribution was not known), and *d* = 0.05 (for precision, corresponding to effect size). The calculation yielded 𝑛 = 1.96² x 0.5(1-0.5) / 0.05² = 384; however, when accounting for a 15% non-response rate, the sample size increased to 440.

Data analysis

The information gathered through the online survey was entered into an Excel sheet, coded, and then analyzed using Statistical Package for Social Sciences (SPSS) Version 26.0 (IBM Corp., Armonk, NY, United States). Counts and percentages were used to describe the data. Tables and figures were used to describe the information. The chi-square test was used to test associations, with a p-value of <0.05 indicating statistical significance.

Ethical considerations

Ethical approval was obtained from the Jazan Heath Ethics Committee via a letter dated March 30, 2023, with approval number REC-44/09/598. A consent question was added to the questionnaire; if the participant refused, the questionnaire link was closed. We ensured that the personal information of participants was preserved and confidentiality maintained.

## Results

A total of 622 participants completed the questionnaire. Most participants were female (58.8%, n = 366). More than half of the participants belonged to the age group of 18-25 years (60.3%, n = 375). Most participants were Saudis (97.3%, n = 605). The majority were single (59.2%, n = 368). Regarding education level, 69.5% (n = 432) of participants had a bachelor's degree. Regarding occupation, most participants (56.6%, n = 352) reported “other” occupations. Around 27.8% (n = 173) of participants were employed. Unemployed participants comprised 10.6% (n = 66) of the sample. Only 1.9% (n = 12) owned private businesses, and 3.1% (n = 19) were retired. Half of the participants lived in cities (50%, n = 311), while 48.4% lived in villages (n = 301); only 1.6% (n = 10) lived in mountainous areas (Table 1).

**Table 1:** Sociodemographic characteristics of the 622 participants

Sociodemographic		N	%
Gender	Male	256	41.2
	Female	366	58.8
Age by year	18-25	375	60.3
	26-35	97	15.6
	36-45	96	15.4
	46-55	42	6.8
	>56	12	1.9
Nationality	Saudi	605	97.3
	Non-Saudi	17	2.7
Marital status	Single	368	59.2
	Married	235	37.8
	Divorced	13	2.1
	Widower	6	1.0
Educational level	Primary	8	1.3
	Secondary	153	24.6
	Higher secondary	15	2.4
	Bachelors	432	69.5
	Other	14	2.3
Occupation	Employed	173	27.8
	Unemployed	66	10.6
	Private business	12	1.9
	Retired	19	3.1
	Other	352	56.6
Residency place	City	311	50
	Village	301	48.4
	Mountainous area	10	1.6

**Figure 1:**
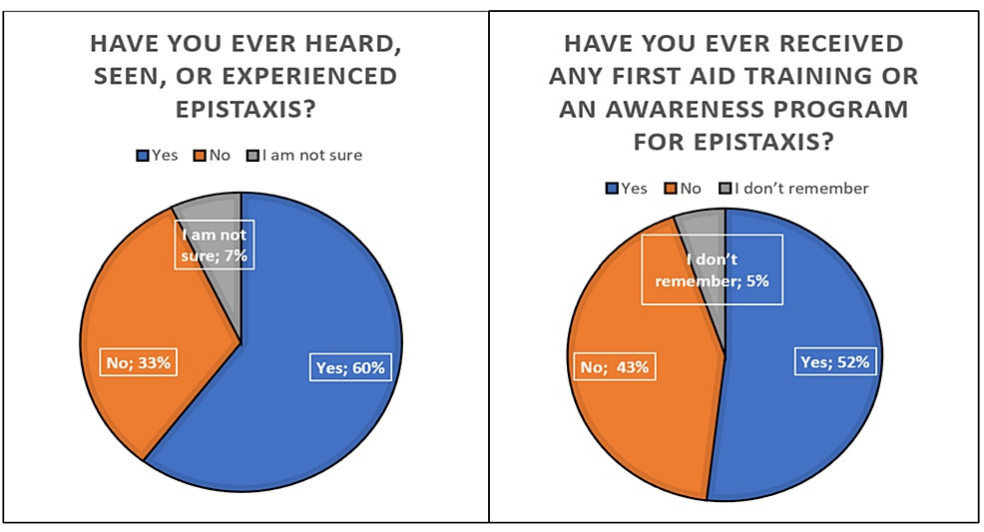
Epistaxis practices among the 622 participants

As shown in Figure 1, regarding epistaxis practices, the majority of participants (60%) had heard of, seen, or experienced epistaxis at some point; however, more than one-third (33%) had not. Only around half (52%) had received any first aid training or awareness regarding epistaxis.

According to the general understanding of epistaxis, the vast majority of respondents (91.8%) correctly identified that epistaxis refers to bleeding from the nose. When asked about the source of epistaxis, 57.6% correctly pointed out that it originates from the inner part of the nose (Table 2).

**Table 2:** Assessment knowledge of first aid for epistaxis *Correct answers

Statement		N	%
Epistaxis bleeding originates from?	Eyes	1	0.2
	Ears	9	1.4
	Nose*	571	91.8
	I don’t know	41	6.6
What is the source of epistaxis?	The inner part of the nose*	358	57.6
	The outer part of the nose	72	11.6
	I don’t know	192	30.9
Do you think the weather is related to epistaxis?	Agree*	362	58.2
	Disagree	42	6.8
	I am not sure	218	35.0
What is the major cause of epistaxis?	Digestive problem	24	3.9
	Trauma and injury*	263	42.3
	I am not sure	335	53.9
What are the most likely risk factors for epistaxis? (Select all that apply)	Local injury to the nasal blood vessels*	124	19.9
	Hypertension*	287	46.1
	Anticoagulant medication*	7	1.1
	Digestive problem	5	0.8
	Prolonged use of non-steroidal anti-inflammatory drugs or steroid nasal sprays*	2	0.3
	Exposure to rain	1	0.2
	Vitamin D deficiency	1	0.2
	I don’t know	193	31.0
What should be the primary step for epistaxis first aid management?	Sitting and leaning head	102	16.4
	Pinching nose	114	18.3
	Applying ice	23	3.7
	All of the above*	254	40.8
	I am not sure	129	20.7
For how long should nose pinching be performed?	2-5 min	240	38.6
	5-10 min*	112	18.0
	15-20 min	13	2.1
	Not sure	257	41.3
Which part of the nose should be pinched with fingers to stop nose bleeding?	Bony	45	7.2
	Cartilage*	235	37.8
	Both	108	17.4
	Not sure	234	37.6
What care should be taken for breathing during nose pinching? Breathing should be?	Maintained through oxygen pump	30	4.8
	Halted during nose pinching	42	6.8
	Allowed through the mouth, avoiding blood swallowing*	264	42.4
	I am not sure	278	44.7
Do you think the icing on the neck region may help manage epistaxis?	Yes*	168	27.0
	No	146	23.5
	Not sure	308	49.5
Do you think cooling or icing can help reduce blood flow from the nose?	Yes*	378	60.8
	No	47	7.6
	Not sure	197	31.7
Icing should be applied to which parts to stop bleeding?	Hand and feet palms	30	4.8
	Nose and back of neck*	274	44.1
	Knees and elbows	5	0.8
	All of the above	45	7.2
	I don’t know	231	37.1
What is the average time in which nose bleeding should stop after first aid management?	10 min*	323	51.9
	20 min	88	14.1
	45 min	10	1.6
	I don’t sure	201	32.3
If the bleeding does not stop within __ minutes, the patient must be moved to __?	10, surgery	46	7.4
	20, hospital*	451	72.5
	30, sleep	12	1.9
	I don’t know	113	18.2

Regarding potential causes and risk factors, there were mixed responses on whether or not the weather is related to epistaxis, with 58.2% agreeing that there is a connection. When asked about the major cause of epistaxis, 42.3% correctly identified trauma and injury as a significant factor. As for the risk factors for epistaxis, hypertension was recognized as a risk factor by 46.1% of respondents, and local injury to the nasal blood vessels by 19.9%. Only a small number of respondents identified anticoagulant medication (1.1%) and prolonged use of non-steroidal anti-inflammatory drugs or steroid nasal sprays (0.3%) as risk factors (Table 2).

Only 40.8% of respondents correctly selected all the steps for first aid management of epistaxis: sitting and leaning the head, pinching the nose, and applying ice. Regarding nose pinching, 37.8% correctly stated that the cartilage part of the nose should be pinched to stop the bleeding. Breathing should be maintained through the mouth to avoid swallowing blood during nose pinching, a fact recognized by 42.4% of respondents. When asked about using cooling or icing to manage epistaxis, 60.8% believed it could help reduce blood flow from the nose, and 27.0% thought that icing the neck region could be beneficial. The correct areas to apply ice - the nose and back of the neck - were identified by 44.1% of the respondents (Table 2).

Regarding the time frame for action, 51.9% correctly stated that nose bleeding should stop within 10 minutes after first aid management. If bleeding does not cease within a specific time, a patient must be moved to the hospital; 72.5% of the respondents correctly identified this time as 20 minutes (Table 2).

**Figure 2:**
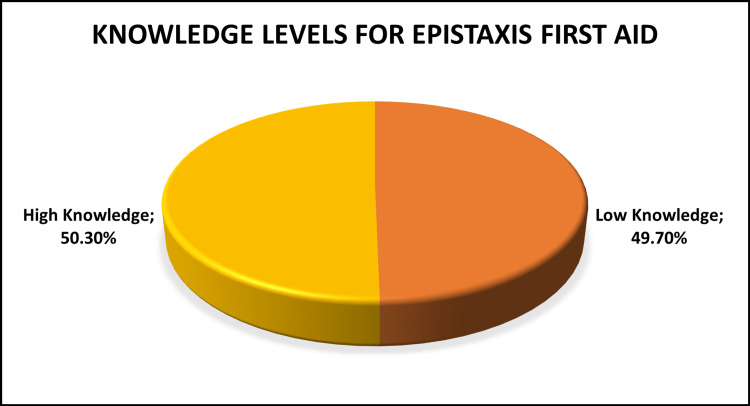
Knowledge levels for first aid for epistaxis

Figure 2 shows the study participants' knowledge levels for first aid for epistaxis. The results indicate that 49.70% of participants had low knowledge of first aid for epistaxis, while 50.30% demonstrated high knowledge of first aid for epistaxis. Knowledge levels were split relatively evenly between the low and high knowledge groups.

Table 3 delineated the significant association between several sociodemographic factors and the hearing, seeing, or experiencing epistaxis; age had been a noteworthy factor, with a significant association (p=0.028). Those aged above 56 had displayed the highest level of hearing, seeing, or experiencing epistaxis, with 75% indicating they had heard, seen, or experienced nosebleeds. In contrast, awareness had dipped to approximately 54.7% in the younger population aged 18-25 years. Nationality was also significantly associated with hearing, seeing, or experiencing epistaxis (p=0.045), with the majority (60.8%) of those familiar with the condition being Saudis. Moreover, a significant association existed between marital status, and hearing, seeing, or experiencing epistaxis (p=0.018). Widows demonstrated the highest at 83.3%, closely followed by married individuals at 67.7%.

**Table 3:** Association between hearing, seeing, or experiencing epistaxis with sociodemographic characteristics and total knowledge. *P < 0.05 (significant). $Fisher exact test.

Sociodemographics		Have you ever heard/seen/experienced epistaxis?			P-value
		Yes, N (%)	No, N (%)	Do not know, N (%)	
Gender	Male	147 (57.4%)	87 (34.0%)	22 (8.6%)	0.303
	Female	229 (62.6%)	115 (31.4%)	22 (6.0%)	
Age (year)	18-25	205 (54.7%)	139 (37.1%)	31 (8.3%)	0.028*
	26-35	61 (62.9%)	28 (28.9%)	8 (8.2%)	
	36-45	71 (74.0%)	22 (22.9%)	3 (3.1%)	
	46-55	30 (71.4%)	10 (23.8%)	2 (4.8%)	
	>56	9 (75.0%)	3 (25.0%)	0 (0.0%)	
Nationality	Saudi	368 (60.8%)	197 (32.6%)	40 (6.6%)	0.045*$
	Non-Saudi	8 (47.1%)	5 (29.4%)	4 (23.5%)	
Marital status	Single	206 (56.0%)	131 (35.6%)	31 (8.4%)	0.018*$
	Married	159 (67.7%)	65 (27.7%)	11 (4.7%)	
	Divorced	6 (46.2%)	6 (46.2%)	1 (7.7%)	
	Widow	5 (83.3%)	0 (0.0%)	1 (16.7%)	
Residency	City	180 (57.9%)	104 (33.4%)	27 (8.7%)	0.199$
	Village	188 (62.5%)	97 (32.2%)	16 (5.3%)	
	Mountainous area	8 (80.0%)	1 (10.0%)	1 (10.0%)	
Education level	Primary	3 (37.5%)	4 (50.0%)	1 (12.5%)	0.001*$
	Secondary	75 (49.0%)	62 (40.5%)	16 (10.5%)	
	High secondary	7 (46.7%)	4 (26.7%)	4 (26.7%)	
	Bachelors	283 (65.5%)	126 (29.2%)	23 (5.3%)	
	Other	8 (57.1%)	6 (42.9%)	0 (0.0%)	
Occupation	Employed	121 (69.9%)	46 (26.6%)	6 (3.5%)	0.016*$
	Unemployed	42 (63.6%)	17 (25.8%)	7 (10.6%)	
	Private business	5 (41.7%)	5 (41.7%)	2 (16.7%)	
	Retired	13 (68.4%)	6 (31.6%)	0 (0.0%)	
	Other	195 (55.4%)	128 (36.4%)	29 (8.2%)	
Total knowledge	Low Knowledge	160 (51.8%)	126 (40.8%)	23 (7.4%)	0.001*
	High Knowledge	216 (69.0%)	76 (24.3%)	21 (6.7%)	

Occupation also significantly affected hearing, seeing, or experiencing epistaxis (p=0.016). Employed individuals had shown the highest hearing, seeing, or experiencing at 69.9%, whereas those engaged in private business reported the lowest at 41.7%. Education level had significantly impacted the hearing, seeing, or experiencing epistaxis (p=0.001). Those holding a bachelor's degree had the highest at 65.5%, while those with only primary education had the lowest at 37.5%. The overall level of knowledge significantly influenced hearing, seeing, or experiencing epistaxis (p=0.001). Approximately 69% of participants possessing a high level of knowledge had heard, seen, or experienced the condition, while this figure dropped to 51.8% among participants with low knowledge.

Hearing, seeing, or experiencing epistaxis had been significantly associated with various sociodemographic factors such as age, nationality, marital status, education level, occupation, and overall knowledge level. However, no significant associations had been found between gender (p=0.303), area of residency (p=0.199), and an individual's familiarity with or experience of epistaxis.

Table 4 shows the association between receiving first aid training or an awareness program for epistaxis and sociodemographic characteristics and total knowledge. There were statistically significant associations between receiving first aid training, and age, gender, marital status, occupation, prior experience with epistaxis, and total knowledge (p<0.05 for all).

**Table 4:** Association between receiving first aid training or an awareness program for epistaxis and sociodemographic characteristics and total knowledge. *P < 0.05 (significant). $Fisher exact test.

Sociodemographics		Have you ever received any first aid training or awareness program for epistaxis?			P-value
		Yes, N (%)	No, N (%)	I do not remember, N (%)	
Age	18-25	220 (58.7%)	133 (35.5%)	22 (5.9%)	0.002*
	26-35	46 (47.4%)	47 (48.5%)	4 (4.1%)	
	36-45	39 (40.6%)	52 (54.2%)	5 (5.2%)	
	46-55	14 (33.3%)	26 (61.9%)	2 (4.8%)	
	>56	4 (33.3%)	8 (66.7%)	0 (0.0%)	
Gender	Male	118 (46.1%)	125 (48.8%)	13 (5.1%)	0.036*
	Female	205 (56.0%)	141 (38.5%)	20 (5.5%)	
Nationality	Saudi	314 (51.9%)	260 (43.0%)	33 (5.1%)	0.323$
	Non Saudi	9 (52.9%)	6 (35.3%)	2 (11.8%)	
Marital status	Single	218 (59.2%)	130 (35.3%)	20 (5.4%)	0.001*$
	Married	95 (40.4%)	129 (54.9%)	11 (4.7%)	
	Divorced	7 (53.8%)	4 (30.8%)	2 (15.4%)	
	Widow	3 (50.0%)	3 (50.0%)	0 (0.0%)	
Residency	City	168 (54.0%)	125 (40.2%)	18 (5.8%)	0.074 $
	Village	150 (49.8%)	136 (45.2%)	15 (5.0%)	
	Mountainous area	5 (50.0%)	5 (50.0%)	0 (0.0%)	
Level of education	Primary	4 (50.0%)	4 (50.0%)	0 (0.0%)	0.177$
	Secondary	78 (51.0%)	63 (41.2%)	12 (7.8%)	
	High secondary	5 (33.3%)	9 (60.0%)	1 (6.7%)	
	Bachelors	232 (53.7%)	182 (42.1%)	18 (4.2%)	
	Other	4 (28.6%)	8 (57.1%)	2 (14.3%)	
Occupation	Employed	75 (43.4%)	91 (52.6%)	7 (4.0%)	0.021*$
	Unemployed	31 (47.0%)	31 (47.0%)	4 (6.1%)	
	Private business	8 (66.7%)	4 (33.3%)	0 (0.0%)	
	Retired	7 (36.8%)	12 (63.2%)	0 (0.0%)	
	Other	202 (57.4%)	128 (36.4%)	22 (6.2%)	
Have you ever heard/seen/experienced epistaxis?	Yes	204 (54.3%)	162 (43.1%)	10 (2.7%)	0.001*
	No	97 (48.0%)	89 (44.1%)	16 (7.9%)	
	I do not know	22 (50.0%)	15 (34.1%)	7 (15.9%)	
Total knowledge	Low Knowledge	133 (43.0%)	149 (48.2%)	27 (8.7%)	0.001*
	High Knowledge	190 (60.7%)	117 (37.4%)	6 (1.9%)	

Younger age groups (18-25 years) were more likely to have received training than older age groups. More females (56.0%) received training compared to males (46.1%). Single individuals (59.2%) were more likely to receive training compared to married individuals (40.4%). Employed individuals were less likely to have received training (43.4%) than retirees and those in other occupations.

Those with prior experience with epistaxis were more likely to have received training (54.3%) than those without prior experience (48.0%). Individuals with high total knowledge about epistaxis were more likely to have received training (60.7%) than those with low knowledge (43.0%). No statistically significant associations were found between receiving training and nationality (p=0.323), residency (p=0.074), or level of education (p=0.177).

## Discussion

Epistaxis, more commonly referred to as a "nosebleed," is a common issue that presents itself in clinical practice and is one of the most prevalent otorhinolaryngologic emergencies [14]. Although often self-limiting, nosebleeds can sometimes escalate into moderate-to-severe forms, requiring prompt medical intervention to control the hemorrhage and prevent any associated complications [3]. Proper first aid measures, including head positioning, nasal compression, and local cooling, can effectively control the bleeding in most epistaxis cases when initiated promptly [15]. However, an insufficiency in public understanding of proper first aid often results in unnecessary visits to the emergency department and hospitalizations [3,16].

This study aimed to assess the knowledge and practices regarding first aid for epistaxis among the population of the Jazan region of Saudi Arabia. The sample for the current study primarily consisted of young adults aged 18-25 years, with a predominance of females. The majority were single Saudi nationals, with a bachelor's degree, and half resided in cities. Despite the global prevalence of epistaxis, only 60% of the participants reported prior experience with the condition, suggesting a possible underreporting due to the perception of nosebleeds as trivial events not requiring medical care [17,18]. Moreover, only half (52%) had received any first aid training for epistaxis management, mirroring findings from other studies conducted in Saudi Arabia and elsewhere [12,19,20]. In Alhassa, Saudi Arabia, 67% had experienced epistaxis before, and 54% had received information about first aid for epistaxis [19]. In Al-Majmaah, Saudi Arabia, 79.86% of people had experienced epistaxis at least once in their lifetime [20]. This lack of public first aid education on common medical emergencies highlights a significant gap that needs to be addressed through large-scale public health initiatives.

The overall knowledge about first aid for epistaxis in this sample was modest, with only 50.3% demonstrating adequate awareness. Most could identify epistaxis as bleeding from the nose (91.8%) and correctly pinpoint its origin in the inner nasal region (57.6%). However, recognizing trauma as the most common cause was low at 42.3%, while medications were rarely identified as risk factors. As for first aid, less than half knew the appropriate steps for head positioning, nasal compression, and icing. Knowledge about the duration of interventions and indications for medical care was better, revealing considerable gaps in understanding the causes, risk factors, and initial management of epistaxis among the public in the Jazan region, a finding that is consistent with past studies [19,20]. In Al-Majmaah, Saudi Arabia, 4.4% and 4.9% experienced it as a result of nasal trauma and fingernail trauma, respectively. Bleeding disorders caused epistaxis in 7.6% of participants, while hypertension was the leading cause in 23% [20]. Plausible reasons for these gaps include inadequate school education, public health campaigns about first aid for common medical emergencies such as epistaxis, and cultural misconceptions around home remedies.

Several sociodemographic parameters were significantly associated with knowledge levels in this study population. Older adults over 56 years of age showed better awareness than younger respondents. This may be attributed to greater cumulative experience managing epistaxis episodes throughout one's lifetime. Married and employed individuals also had higher knowledge, possibly due to greater family responsibilities. Higher education status is expectedly correlated with greater knowledge, highlighting the importance of literacy. People with higher education levels tend to acquire more information and have better access to resources, which can contribute to their overall knowledge and awareness.

An interesting finding was that experiencing epistaxis was more common among Saudi nationals than non-Saudis, possibly linked to the prevalence of cultural and traditional home remedies for epistaxis in the native population. Widows also reported greater experience with epistaxis, likely due to being more actively involved in family caregiving roles after losing a spouse [21]. These findings provide vital insights into the demographic segments that should be targeted to improve awareness about first aid for epistaxis, such as youth and socially isolated groups. One way to achieve this is through educational campaigns and awareness programs focusing on these populations. Social media can also be a valuable tool for disseminating information and supporting these groups [22].

Significant associations were observed between first aid training status and factors such as female gender, youth, single status, and prior epistaxis knowledge. Females, often the primary caregivers in domestic settings, might be motivated to acquire first aid skills through training. Younger, unmarried adults likely have more free time and willingness to attend such programs. Prior experience or knowledge about epistaxis would logically spark interest in further self-education through first aid courses [23-25].

This study offers valuable insights into the knowledge gaps and demographic determinants pertaining to awareness about first aid for epistaxis among adults in the Jazan region of Saudi Arabia. The findings underscore the need for large-scale community education initiatives tailored to diverse societal groups. These could take the form of awareness programs emphasizing the role of caregivers and family protectors to attract housewives and married women or public campaigns, highlighting the importance of first aid skills for job prospects to benefit unemployed young adults. Innovative social media-based apps and e-learning platforms could be used to engage technologically savvy youth.

Furthermore, the study suggests including first aid training in school and college curricula and offering workplace training opportunities, especially for blue-collar migrant workers. Government health agencies should fund public health campaigns tailored to local contexts, including rural areas. The strategic use of mass media such as television, radio, and social networks can facilitate broader outreach. The recently launched Saudi First Aid Platform is a step in the right direction.

Support groups and non-profit sector engagement could also help facilitate training for the elderly, women, and economically disadvantaged groups. Policy-level changes to increase the accessibility of approved first aid courses and the availability of trained professionals for public education are imperative. Future studies should explore the barriers to implementing proper first aid despite adequate knowledge. Addressing these barriers can help translate awareness into practice and improve health outcomes.

Study limitations

This study has several limitations. The convenience sampling method and overrepresentation of younger, more educated females may limit the generalizability of the findings. The study relied on self-reported data, introducing potential biases such as social desirability or recall errors. As a cross-sectional analysis, it cannot establish causality between variables. Additionally, confounding factors such as prior education, access to information, and socioeconomics were not measured. Actual first aid practices for epistaxis were not observed; only self-reported attitudes and knowledge were observed.

## Conclusions

The study on the general population in the Jazan region of Saudi Arabia revealed varying knowledge, attitudes, and practices concerning first aid for epistaxis. While most participants knew of epistaxis, only half had received first aid training. Understanding causes, risk factors, and appropriate first aid steps was limited. There was a near-equal divide between participants with low and high knowledge levels.

There were significant associations between several sociodemographic factors, experiences of epistaxis, and the receipt of first aid training. Younger individuals, females, singles, students, and those with prior epistaxis experience were likelier to have received initial training. Interestingly, participants with higher knowledge levels were also more likely to have received training.

The findings indicate a need for more comprehensive public awareness campaigns and first aid education programs in the Jazan region, mainly targeting older individuals, males, married people, non-students, and those without prior epistaxis experience. Improving the population's knowledge regarding proper first aid for epistaxis could help mitigate its effects and complications. Future research should explore efficient educational interventions to enhance the general population's understanding of first aid for epistaxis.
